# Transcriptomic Analysis of Naïve Human Embryonic Stem Cells Cultured in Three-Dimensional PEG Scaffolds

**DOI:** 10.3390/biom11010021

**Published:** 2020-12-28

**Authors:** Christina McKee, Christina Brown, Shreeya Bakshi, Keegan Walker, Chhabi K. Govind, G. Rasul Chaudhry

**Affiliations:** 1 Department of Biological Sciences, Oakland University, Rochester, MI 48309, USA; cmmckee@oakland.edu (C.M.); brown3@oakland.edu (C.B.); sbakshi@umich.edu (S.B.); keeganjwalker22@gmail.com (K.W.); govind@oakland.edu (C.K.G.); 2 OU-WB Institute for Stem Cell and Regenerative Medicine, Rochester, MI 48309, USA

**Keywords:** three-dimensional culture, self-assembling PEG scaffolds, naïve embryonic stem cells, signaling pathways, mechanotransduction

## Abstract

Naïve human embryonic stem cells (ESCs) are characterized by improved viability, proliferation, and differentiation capacity in comparison to traditionally derived primed human ESCs. However, currently used two-dimensional (2-D) cell culture techniques fail to mimic the three-dimensional (3-D) in vivo microenvironment, altering morphological and molecular characteristics of ESCs. Here, we describe the use of 3-D self-assembling scaffolds that support growth and maintenance of the naïve state characteristics of ESC line, Elf1. Scaffolds were formed via a Michael addition reaction upon the combination of two 8-arm polyethylene glycol (PEG) polymers functionalized with thiol (PEG-8-SH) and acrylate (PEG-8-Acr) end groups. 3-D scaffold environment maintained the naïve state and supported the long-term growth of ESCs. RNA-sequencing demonstrated significant changes in gene expression profiles between 2-D and 3-D grown cells. Gene ontology analysis revealed upregulation of biological processes involved in the regulation of transcription and translation, extracellular matrix organization, and chromatin remodeling in 3-D grown cells. 3-D culture conditions also induced upregulation of genes associated with Wnt and focal adhesion signaling, while p53 signaling pathway associated genes were downregulated. Our findings, for the first time, provide insight into the possible mechanisms of self-renewal of naïve ESCs stimulated by the transduction of mechanical signals from the 3-D microenvironment.

## 1. Introduction

Embryonic stem cells (ESCs) are pluripotent cells capable of unlimited self-renewal and differentiation into all three germ layers [1,2], making them an attractive source for the development of in vitro disease models and drug discovery as well as tissue engineering and cellular therapies [3,4,5]. These applications require stringent conditions for characterization and expansion as well as differentiation of cells into homogenous populations of progenitors or derivatives. However, these processes are hindered both by the limited understanding of pluripotent cell biology as well as current culture techniques [6,7].

Traditionally, human ESCs are derived from the inner cell mass of a preimplantation blastocyst, exhibiting growth characteristics similar to mouse epiblast stem cells (EpiSCs) in vitro [8]. In contrast, mouse ESCs, isolated at an earlier developmental stage, show improved clonal growth and viability following single-cell dissociation and are capable of contributing to blastocyst chimeras [9]. Based on their divergent characteristics in vitro, two distinct stages of pluripotency were proposed, an early naïve state (mouse ESCs) and a primed state (human ESCs and mouse EpiSCs) [10].

Naïve pluripotency affords certain advantages over traditionally derived primed ESCs, including improved proliferation and developmental potential as well as single-cell cloning efficiency and genome editing [11,12]. Consequently, a great deal of effort has been focused on both the reprogramming and establishment of naïve human ESC lines [9,13,14].

Several reports described the successful culture of human ESCs in a naïve pluripotent state using ectopic expression of select genes and inhibitor cocktails [11,15,16,17,18,19]. Recently, improved culture conditions led to the derivation of naïve human ESC lines directly from six to eight-cell embryos [20,21,22]. This has led to an increased understanding of the importance of defined culture media conditions for the modulation of cell signaling and maintenance of pluripotency in vitro.

However, ESC culture practices are still antiquated, relying on adherence to two-dimensional (2-D) tissue-culture polystyrene plates often coated with natural or synthetic substrates mimicking extracellular matrix (ECM) components for the propagation of cells [6]. Generally, 2-D culture conditions fail to mimic the native three-dimensional (3-D) microenvironments that are responsible for the regulation of cell fate in vivo, allowing for dynamic spatial interactions between cells, ECM components, and gradients of soluble factors via biochemical, mechanical, and structural stimuli [23,24,25]. 2-D culture fails to accurately reproduce the physiology of the embryo [26], resulting in the spontaneous differentiation of ESC colonies in vitro [27,28].

Therefore, the development of 3-D culture techniques via the incorporation of bioinductive materials has gained traction in recent years [6]. Natural biomaterials such as collagen type 1, gelatin, alginate, and hyaluronic acid have been studied for the 3-D propagation of human ESCs [29,30,31,32,33]. However, these materials have been shown to induce biological signaling and variability in culture [34]. In contrast, inert synthetic biomaterials can be more easily modified, allowing for tunable mechanical and biophysical properties as well as minimizing batch-to-batch variation [35]. Therefore, synthetic biomaterials may prove to be ideal for the development of the defined 3-D culture conditions needed for the propagation of human ESCs. We have previously demonstrated 3-D culture of naïve mouse ESCs [36] and primed human ESCs [37] using hydrogel scaffolds that resulted in modulation of gene expression and maintenance of pluripotent cells. In this study, we investigated the 3-D culture of naïve human ESCs (Elf1 cells) using self-assembling scaffolds comprised of two synthetic 8-arm polyethylene glycol (PEG) polymers functionalized with thiol (PEG-8-SH) and acrylate end groups (PEG-8-Acr). 3-D culture in the PEG-8-SH/PEG-8-Acr scaffolds supported ESC self-renewal and pluripotency. To determine the effect of culture in the PEG-8-SH/PEG-8-Acr scaffolds on the maintenance of pluripotency, we compared changes in the transcriptomic profile of naïve ESCs grown in 2-D and 3-D culture conditions. The improved understanding of the molecular functions and pathways associated with continued self-renewal of naïve ESCs enriched in 3-D culture may lead to improvements in in vitro culture techniques, thus promoting their use in basic and applied applications.

## 2. Materials and Methods

### 2.1. Maintenance of Naïve ESCs in 2-D Culture

Elf1 cells, a naïve human ESC line isolated from an 8-cell human embryo, were procured from Wicell (Madison, WI, USA) and maintained according to a previously published protocol [22]. Briefly, Elf1 cells were grown adherent to mouse embryonic feeder (MEF) coated 6-well plates and incubated in a medium containing KnockOut/F12 DMEM with GlutaMax (Life Technologies, Carlsbad, CA, USA) with 20% KnockOut serum replacement (Life Technologies), 1 mM sodium pyruvate (Life Technologies), 0.1 mM 2-Mercaptoehtanol (Life Technologies), 1% non-essential amino acids (Life Technologies), and 0.2% penicillin-streptomycin solution (Life Technologies), and supplemented with 12 ng/mL FGF2 (Prospec, Ness Ziona, Israel), 1.5 μM CHIR99021 (Cayman Chemical, Ann Arbor, MI, USA), 0.4 μM PD03296501 (Caymen Chemical) and 0.01 μg/mL human LIF (Prospec). Prior to encapsulation in 3-D scaffolds, cells were transitioned to feeder-free culture in Matrigel-coated 6-well plates (Fisher Scientific, Pittsburgh, PA, USA).

### 2.2. Self-Assembly of 3-D Scaffolds Via Thiol-Michael Addition Reaction for Encapsulation of Cells

**Figure 1:**
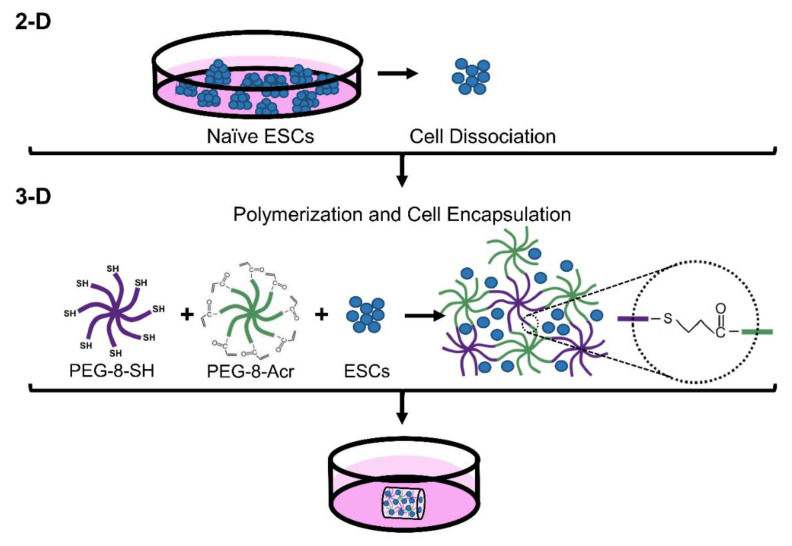
Encapsulation and 3-D culture of naïve human embryonic stem cells (ESCs) in self-assembling scaffolds. Elf1 cells grown in 2-D culture conditions were dissociated into single cells and mixed with self-assembling end-functionalized polymers, polymers functionalized with thiol (PEG-8-SH) and acrylate (PEG-8-Acr). The polymer/cell mixture was then transferred to a syringe mold and allowed to form 3-D scaffolds, encapsulating the cells. The scaffolds were then transferred to 24-well plates containing culture medium.

8-arm PEG polymers functionalized with thiol and acrylate end groups were purchased from JenKem Technology USA (Plano, TX, USA) and stored in the dark at −20 °C. PEG-8-SH and PEG-8-Acr polymers were combined to facilitate self-assembly, as previously described [37]. Briefly, PEG-8-SH and PEG-8-Acr were separately dissolved in culture media at 2.5 *w/v*% polymer concentration and mixed thoroughly at a 1:1 molar ratio in the presence of oxygen for polymerization via a thiol-Michael addition reaction. The schematic of scaffold formation is depicted in Figure 1. For 3-D culture, ESCs were dissociated into single cells using Accutase (Life Technologies), pelleted at 5 × 10^6^ cells/mL, resuspended in the PEG-8-SH/PEG-8-Acr polymer mixture, and transferred to syringe molds for polymerization, forming 100 µL scaffolds. Following scaffold formation, scaffolds were placed in 24-well culture plates (Fisher Scientific), supplemented with culture medium, and incubated at 37 °C and 5% CO_2_. The medium was changed daily or as needed.

### 2.3. Cell Proliferation of 3-D Grown ESCs

The proliferation of cells grown under 3-D culture conditions was monitored by phase-contrast microscopy, and colony size was quantified by determining the average mean diameter using ImageJ software. Cell growth was also quantified at various time points via direct cell counts using a hemocytometer and an MTT proliferation assay. Briefly, samples were prepared in triplicate experiments and treated with 5 mg/mL MTT reagent (Sigma, St. Louis, MO, USA), incubated at 37 °C for 4 h, and subsequently treated with isopropanol/HCL (15:1) to solubilize the formazan product produced by viable cells [38]. The absorbance of the solubilized formazan was then measured at 570 nm using an Epoch microplate spectrophotometer (BioTek, Winooski, VT, USA), and the background absorbance was subtracted from all experimental values.

### 2.4. Teratoma Formation Assay

The pluripotency of 3-D grown cells was assessed by teratoma formation assays performed in triple experiments. ESCs (1 × 10^6^) were dissociated into single cells following Accutase treatment, resuspended in PBS, mixed with an equal volume of Matrigel (BD Biosciences, San Jose, CA, USA), and injected subcutaneously into the flanks of 4-week-old immunocompromised Fox Chase Severe Combined Immunodeficiency (SCID) Beige mice (Charles River, Wilmington, MA, USA) using a Hamilton syringe. Animals were monitored daily and humanely euthanized by CO_2_ overdose following teratoma formation at 10–12 weeks post-injection. Explanted teratomas were either fixed for histological analysis or flash frozen for RNA isolation and assessed by immunohistochemistry and quantitative real time-polymerase chain reaction (qRT–PCR) for germ layer marker expression, respectively. All procedures involving animals were approved by the Institutional Animal Care and Use Committee of Oakland University (IACUC protocol number: 17,031).

### 2.5. Gene Expression Analysis Using qRT–PCR

Total cellular mRNA was isolated from cells grown in 2-D and 3-D culture conditions using the GeneJET RNA purification kit (Thermo Fisher Scientific). For analysis of germ layer markers, total RNA was extracted from teratoma tissue (100–250 mg) using RNeasy midi kit (Qiagen, Germantown, MD, USA) per the manufacturer’s instructions. cDNA was synthesized using the iScript kit (Bio-Rad, Hercules, CA, USA). qRT–PCR was performed using SsoAdvanced SYBR Green Supermix (Bio-Rad) and the CFX96 real-time PCR system. Triplicate reactions were normalized to reference genes, *HMBS* and *GAPDH.* Primers (IDT Technologies, Coralville, IA, USA) used in this study are presented in Table 1.

### 2.6. RNA-Sequencing (RNA-seq)

Total RNA from 2-D and 3-D grown cells was isolated as described. RNA was quantified and qualified using an Agilent2100 Bioanalyzer (Agilent Technologies, Palo Alto, CA, USA) and Qubit Assay (Life Technologies). RNA with an RNA integrity number (RIN) of 10 was used as input material for library preparation. cDNA libraries were prepared using the KAPA RNA HyperPrep Kit with RiboErase (HMR) (Kapa Biosystems, Wilmington, MA, USA) according to the manufacturer’s protocol [39] and sent for RNA-seq transcriptome analysis.

GENEWIZ (South Plainfield, NJ, USA) performed 2 × 150 bp paired-end read sequencing on the Illumina NovaSeq/HiSeq. An average of 47 million reads was obtained for each sample. Fragments were mapped to reference human genome assembly hg38 (with an average mapping efficiency of 94%), and differential gene expression analysis was performed using the Galaxy platform (https://usegalaxy.org/) [40] as detailed below. RNA-Seq analyses were performed on two independent biological replicates. RNA-seq data were deposited into GEO with an accession number of GSE161306.

### 2.7. Bioinformatic Analysis

The quality of the raw reads was assessed using FastQC toolkit v0.72 [41]. Low-quality reads and adapters were trimmed using Trim Galore! v0.6.3 [42]. HiSat2 v2.1.0 [43] was then used for mapping the raw reads to the reference genome human genome assembly hg38. The expression for each gene was evaluated using featureCounts v1.6.4 [44] based on annotation from GENCODE release 30 [45]. The raw counts were then normalized, and differential gene expression was determined using DESeq2 v2.11.40.6 [46]. Genes with an adjusted *p*-value or Benjamini–Hochberg false discovery rate (FDR) adjustment < 0.05 were considered significant unless otherwise specified.

Biological significance and functional assessment of genes differentially expressed between 2-D and 3-D culture conditions were analyzed by gene ontology (GO) assessment. Protein analysis through evolutionary relationships (PANTHER) [47] and the Kyoto Encyclopedia of Genes and Genomes (KEGG) pathway [48] databases were used to analyze biological pathways associated with differentially expressed genes (DEGs). In addition, enrichment of DEGs associated with cellular compartment, molecular function and biological processes was also performed using Enrichr [49,50]. Significance was defined by the recommended *p*-value < 0.05. Heatmaps were generated using Heatmapper (http://www.heatmapper.ca/) [51].

### 2.8. Immunohistochemical Analysis

Protein expression of germ layer markers was investigated by fluorescent immunohistochemical staining. Teratomas were fixed overnight in 4% paraformaldehyde and embedded in O.C.T solution for cryosectioning into 10 μm sections. For immunohistochemical analysis, cryosections were permeabilized with 0.5% Triton X-100 (Sigma) for 10 min and blocked with 2% BSA (Sigma) for 1 h at room temperature. Samples were incubated overnight at 4 °C with primary antibodies (1:100 dilution) against GATA4 (sc-25310, Santa Cruz Biotechnology, Santa Cruz, CA), BRACHYURY (sc-20109, Santa Cruz), and TUJ1 (sc-58888, Santa Cruz) representing endoderm, mesoderm, and ectoderm germ layers, respectively. Primary antibody-treated samples were then washed with PBS, stained with anti-rabbit Alexa Fluor 488 (A32731, Thermo Fisher Scientific) or anti-mouse Cy3-labeled IgG (072-01-18-06, KPL, Gaithersburg, MD, USA) secondary antibodies at 1:200 dilution for 2 h at room temperature, and counterstained with DAPI (1 mg/mL; Life Technologies). The stained samples were visualized by using confocal microscopy.

### 2.9. Statistical Analysis

Data are presented as mean ± standard error of the mean (SEM). One-way ANOVA analysis was performed to determine statistical significance, and data were analyzed for unequal variances using post hoc tests for multiple comparisons. Results with a *p*-value less than 0.05 were considered to be significant with * *p* < 0.05 and ** *p* < 0.01. All analyses were performed using SPSS version 26 (IBM Inc., Armonk, NY, USA).

## 3. Results

### 3.1. Proliferation and Characteristics of Pluripotent ESCs Grown in 3-D Self-Assembling Scaffolds

**Figure 2:**
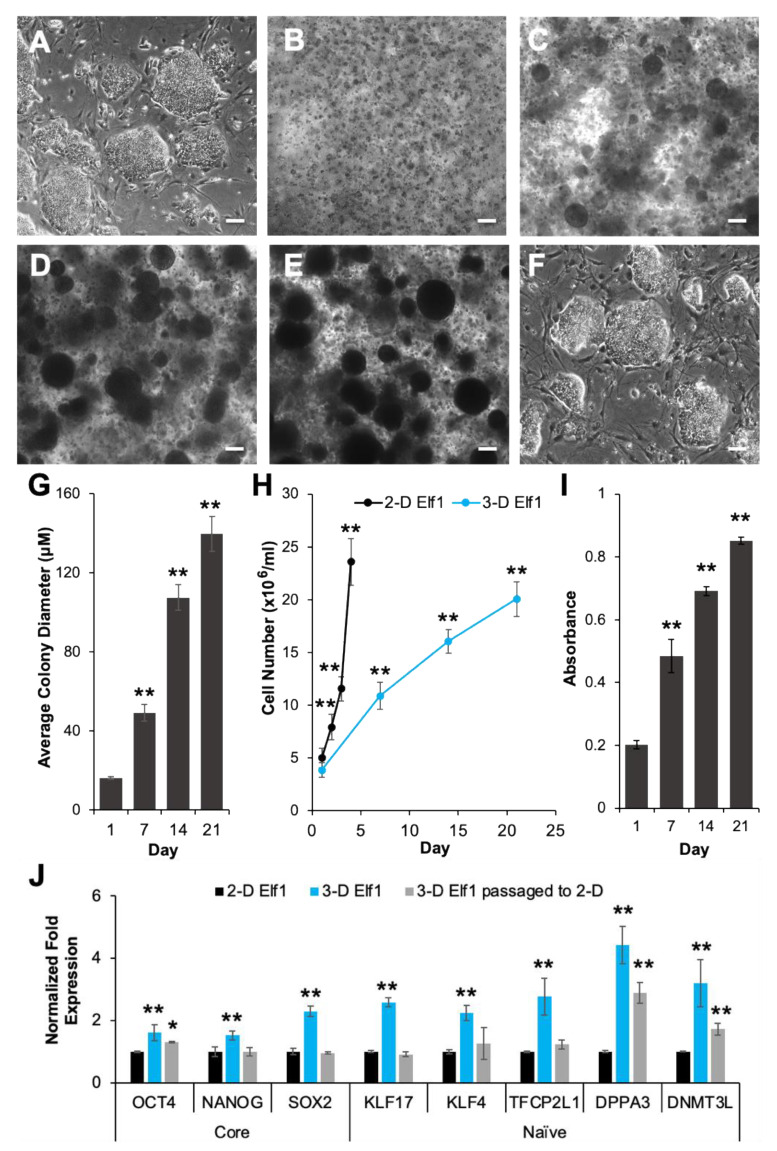
Comparison of naïve ESCs grown in 2-D and 3-D culture conditions. (**A**) Light micrographs (LM)showing the morphology of naïve human ESCs (Elf1) cells grown in 2-D conditions, (**B**–**E**) LM showing the morphology of Elf1 cells grown in 3-D PEG-8-SH/PEG-8-Acr scaffolds for 0, 7, 14 and 21 days, respectively. (**F**) LM of Elf1 cells grown in 3-D and then subcultured back to 2-D culture conditions displayed undifferentiated morphology and clonal growth comparable to the initial 2-D grown cells. All scale bars represent 100 μm. (**G**) Quantification of colony size of Elf1 cells grown in 3-D scaffolds was determined using ImageJ software and expressed as average diameter (μm) ± SEM. (**H**) Growth of Elf1 cells in 2-D and 3-D culture conditions was assayed by direct counts at various time points using a hemocytometer. Data are presented as cell number (×10^6^ cells/mL) ± SEM. (**I**) Quantitative determination of ESC proliferation by MTT assay. Results were expressed as the absorbance ± SEM with a significant increase in cell number. (**J**) Expression of core and naïve pluripotency markers in Elf1 cells grown in 2-D and 3-D culture conditions as well as 3-D grown cells passaged to 2-D culture as determined by qRT–PCR. Results were expressed as the fold expression ± SEM normalized to reference genes *HMBS* and *GAPDH* (* *p* ≤ 0.05 and ** *p* ≤ 0.01).

We first investigated the encapsulation and proliferation of Elf1 cells in 3-D self-assembling scaffolds by light microscopy and MTT analysis (Figure 2). The results presented in Figure 2A show that Elf1 cells displayed compact clonal morphology and growth, consistent with naïve ESCs grown in 2-D culture conditions. When Efl1 cells were encapsulated in 3-D scaffolds, they proliferated with a distinctly progressive increase in the size of dark and densely packed colonies (Figure 2B–E). The size of encapsulated colonies increased during culture, reaching an average of 140 μM by day 21 of 3-D culture (Figure 2G). When subcultured back to 2-D culture conditions, these 3-D grown cells maintained their undifferentiated clonal morphology, indistinguishable from the initial cells (Figure 2F). The growth of encapsulated cells was also analyzed by direct cell counts, as shown in Figure 2H. When grown in 2-D culture conditions, Elf1 cells grew rapidly, requiring routine passaging every 3 to 4 days to minimize spontaneous differentiation. In comparison, 3-D grown cells proliferated more slowly, with a similar fold increase observed following 21 days of culture without noticeable differentiation. The proliferation of cells encapsulated in the 3-D scaffolds was further confirmed via quantitative MTT analysis, which showed a steady and significant increase in cell growth over the 21-day period (Figure 2I). Characterization of 3-D grown Elf1 cells depicted in Figure 2J show upregulation in the expression of core pluripotent markers, *OCT4*, *NANOG*, and *SOX2*, which increased 1.6-, 1.5-, and 2.3-fold, respectively on day 21 of culture. Interestingly, similar but more pronounced increases were observed in the expression of naïve markers, *KLF17*, *KLF4*, *TFCP2L1*, *DPPA3*, and *DNMT3L*, which increased by 2.6-, 2.2-, 2.7-, 4.4-, and 3.2-fold, respectively. A precipitous decrease in the expression levels of most of the core and naïve genes was observed upon subculturing the 3-D grown Elf1 cells under 2-D culture conditions. The expression levels decreased to levels that were similar to that of the initial 2-D grown cells. Although the expression of *OCT4*, *DPPA3*, and *DNMT3L* genes remained relatively high, they gradually decreased to normal levels upon repeated passaging under 2-D culture conditions (data not shown). Together these results suggest that the 3-D culture conditions effectively maintained the pluripotent growth of Elf1 cells.

**Figure 3:**
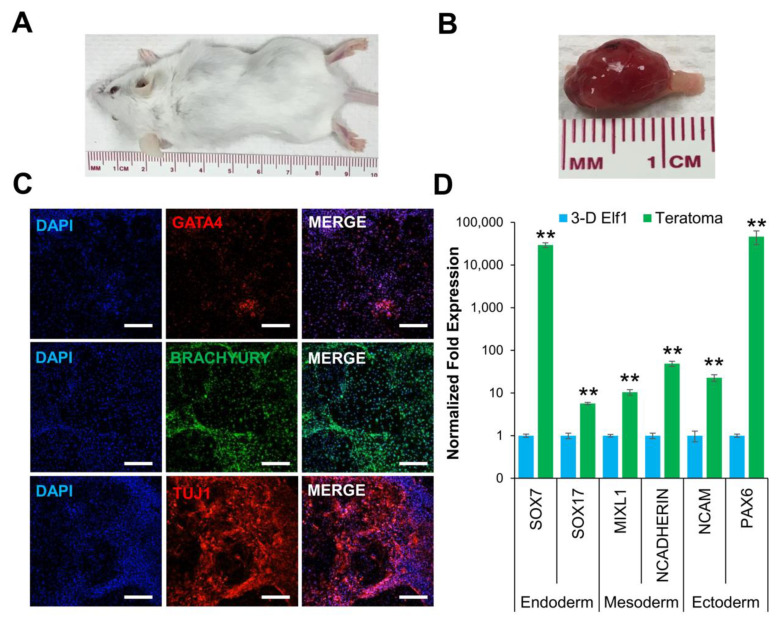
3-D cultured naïve ESCs produced teratomas in SCID-beige mice. (**A**) Tumor growth was observed in all mice injected with 3-D grown Elf1 cells (*n* = 3). (**B**) Explanted tumor at 4 weeks showed well-circumscribed lobular morphology consistent with teratoma formation. (**C**) Confocal images (10×) depict the presence of GATA4, BRACHYURY, and TUJ1 protein expression representing endoderm, mesoderm, and ectoderm, respectively. All scale bars represent 100 μm. (**D**) Gene expression analysis by qRT–PCR showed that teratomas expressed *SOX7*, and *SOX17* (endoderm)*, MIXL1*, and *N-CADHERIN* (mesoderm), and *NCAM,* and *PAX6* (ectoderm). Results are expressed as the fold expression ± SEM normalized to reference genes *HMBS* and *GAPDH* (** *p* ≤ 0.01).

These results were then validated by assessing the differentiation potential of 3-D grown Elf1 cells both in vitro (data not shown) and in vivo by teratoma formation assay. The explanted teratomas were analyzed by immunohistochemical staining and qRT–PCR (Figure 3C,D). The results show positive expression of GATA4, BRACHYURY, and TUJ1 proteins, representative of endoderm, mesoderm, and ectoderm tissue, suggesting that 3-D grown Elf1 cells differentiated into all three germ layers in vivo. Transcriptional analysis of teratomas also showed expression of germline-specific markers, *SOX7* and *SOX17* (endoderm), *MIXL1* and *N-CADHERIN* (mesoderm), as well as *NCAM* and *PAX6* (ectoderm). Overall, these results confirmed that 3-D culture conditions maintained the characteristics of Elf1 cells and upregulated the expression of core naïve genes, suggesting that the self-assembling scaffolds provided a supportive microenvironment for pluripotency.

### 3.2. Transcriptomic Analysis of Naïve ESCs Grown in 2-D and 3-D Culture Conditions

**Figure 4:**
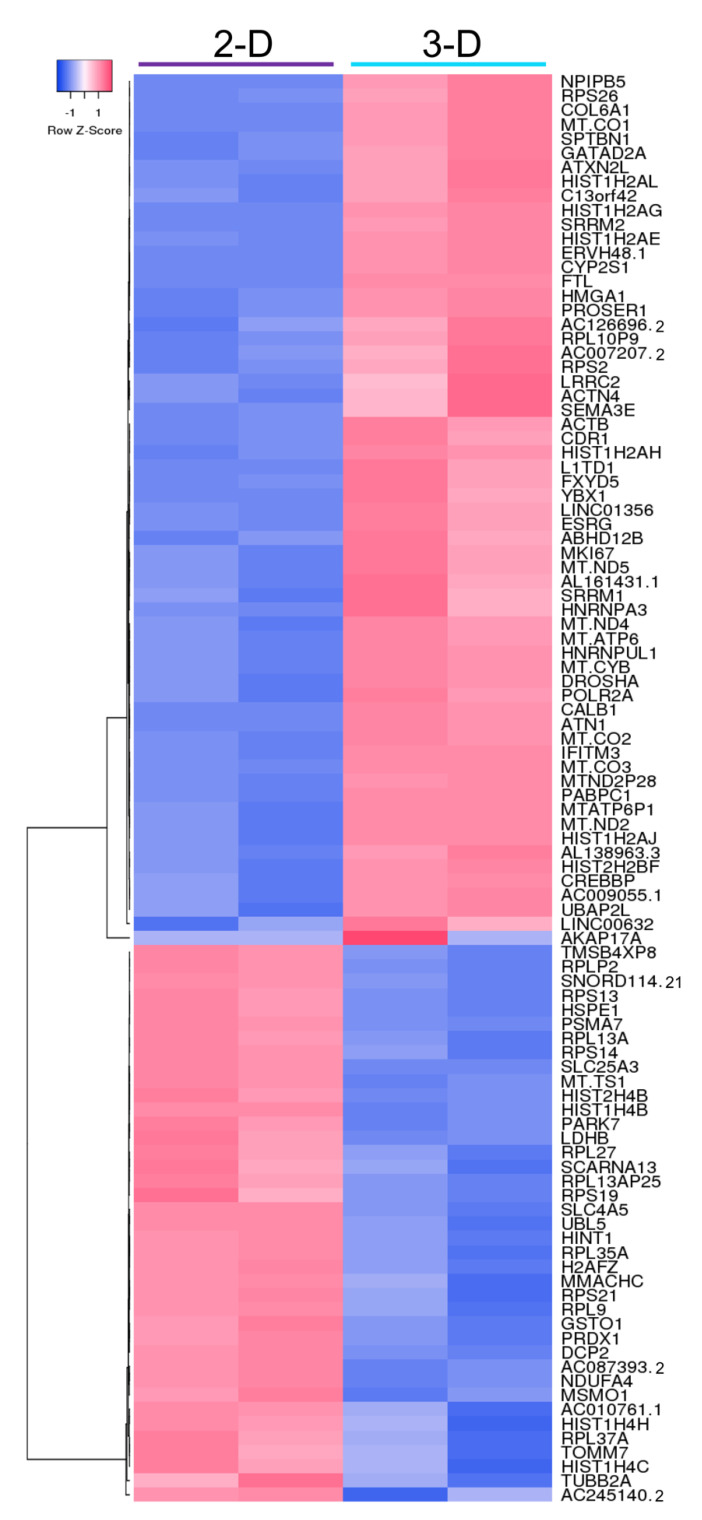
Heatmap showing raw z-scores of RNA-seq log2 transformed values of the top 100 differentially expressed genes in 2-D and 3-D cultured Elf1 cells. Up- and downregulated genes are red and blue, respectively.

Many studies have shown that 3-D cell culture conditions can modulate gene expression via biological and/or mechanical signals [23,24]. To investigate the molecular level differences in Elf1 grown in 2-D and 3-D culture conditions, RNA-seq was performed. Differential expression analysis of the RNA-seq data identified 802 differentially expressed genes (DEGs) at FDR < 0.01 and 1290 DEGs at FDR < 0.05, with 489 and 792 upregulated genes in 2-D and 3-D grown cells, respectively. The clear distinction of the transcriptomic profile between culture conditions was evident in the heatmap depicting the top 100 DEGs (FDR < 4.0 × 10^−9^), of which 39 genes were upregulated in 2-D grown cells and 61 genes were upregulated in 3-D grown cells (Figure 4).

### 3.3. Functional Analysis of DEGs Using Gene Ontology (GO) and Enrichment Analysis

**Figure 5:**
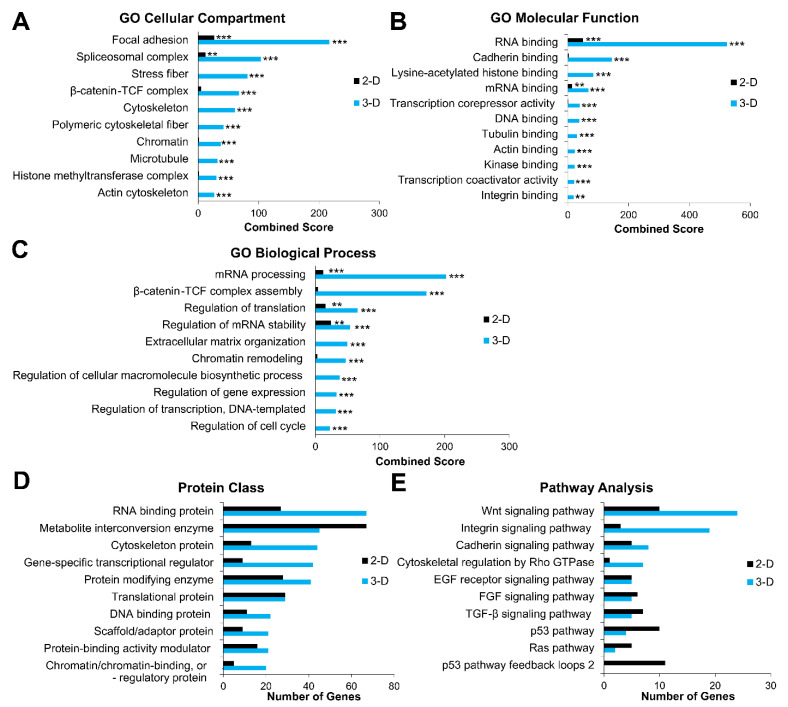
Gene ontology analysis of differentially expressed genes in naïve ESCs cultured under 2-D and 3-D conditions. (**A**–**C**) Enrichr and (**D**,**E**) protein analysis through evolutionary relationships (PANTHER) analysis of differential gene expression with Benjamini–Hochberg false discovery rate (FDR) < 0.05 as determined by DESeq2 analysis. (**A**–**C**) Gene ontology (GO) term enrichment analysis depicting upregulated differentially expressed genes (DEGs) associated with the cellular compartment, molecular function, and biological processes, respectively. The *x*-axis represents the combined score (*p*-value multiplied by the z-score) generated by Enrichr (** *p* ≤ 0.01, and *** *p* ≤ 0.001). (**D**,**E**) Upregulated protein classes and pathways associated with 2-D in 3-D cultured Elf1 cells according to PANTHER analysis, respectively. The *x*-axis represents the number of genes associated with each category.

GO and pathway analysis of genes with an FDR < 0.05 were performed using Enrichr and PANTHER (Figure 5). Comparative enrichment analysis of Elf1 cells grown in 2-D and 3-D culture conditions performed using Enrichr identified DEGs associated with cellular compartment, molecular function, and biological process ontologies (Figure 5A–C). In the cellular compartment category, 3-D grown cells were found to be highly enriched for GO terms, including focal adhesion (GO:0005925), spliceosomal complex (GO:0005681), stress fiber (GO:0001725), β-catenin-TCF complex (GO:1990907), cytoskeleton (GO:0005856), polymeric cytoskeleton fiber (GO:0099513), chromatin (GO:0000785), microtubule (GO:0005874), histone methyltransferase complex (GO:0035097), and actin cytoskeleton (GO:0015629) (Figure 5A). While upregulated DEGs in 2-D grown cells were only significantly enriched in the cellular compartment categories of focal adhesion and spliceosomal complex, with much lower combined scores when compared to 3-D grown cells.

GO terms for molecular function of DEGs upregulated in 3-D culture showed significant enrichment in RNA-binding (GO:0003723), cadherin binding (GO:0045296), lysine-acetylated histone-binding (GO:0070577), mRNA-binding (GO:0003729), transcription corepressor-binding (GO:0003714), DNA-binding (GO:0003677), tubulin-binding (GO:0015631), actin-binding (GO:0003779), kinase-binding (GO:0019900), transcription coactivator activity (GO:0003713), and integrin-binding (GO:0005178) (Figure 5B). For these functions, 2-D grown cells showed enrichment of DEGs in categories of RNA-binding and mRNA-binding at levels significantly lower than that of 3-D grown cells.

When gene products were classified according to biological process GO terms, 3-D grown cells exhibited significant enrichment in pathways/processes associated with mRNA processing (GO:0006397), β-catenin-TCF complex assembly (GO:1904837), regulation of translation (GO:0006417), regulation of mRNA stability (GO:0043488), extracellular matrix organization (GO:0030198), chromatin remodeling (GO:0006338), regulation of cellular macromolecular biosynthetic process (GO:2000112), regulation of gene expression (GO:0010468), regulation of DNA-templated transcription (GO:0006355), and regulation of cell cycle (GO:0051726) (Figure 5C). Of these biological processes, 2-D grown cells displayed significant upregulation of DEGs in mRNA processing, regulation of translation, and regulation of mRNA stability, with a slight but not significant increase in β-catenin-TCF complex assembly and chromatin remodeling categories.

GO analysis was also performed using PANTHER to identify protein classes and pathways upregulated in DEGs in 2-D and 3-D culture conditions, with 431 (out of 484) genes and 744 (out of 812) genes mapped to the PANTHER database, respectively. Predominant protein classes expressed in 3-D grown cells include RNA-binding protein (PC00031), followed by cytoskeleton protein (PC00085), gene-specific transcriptional regulator (PC00264), protein modifying enzyme (PC00260), DNA-binding protein (PC0009), scaffold/adaptor protein (PC00226), protein-binding activity modulator (PC00095), and chromatin/chromatin-binding or -regulatory protein (PC00077) (Figure 5D). On the other hand, a higher number of DEGs were associated with metabolite interconversion enzyme (PC00262) in 2-D cultured cells, and the total number of DEGs associated with translational proteins (PC00263) were the same in cells grown both 2-D and 3-D conditions.

To investigate the effect of 2-D and 3-D culture conditions on cell signaling, pathway analysis of the DEGs was performed. A higher number of genes were associated with Wnt (P00057), integrin (P00034), cytoskeleton regulation by Rho GTPase (P00016), and cadherin (P00012) signaling in 3-D grown cells (Figure 5E). While the number of genes associated with FGF (P00021) and TGF-β (P00052) signaling pathways were slightly higher in 2-D compared to 3-D cultured cells, a greater increase was observed in Ras (P04393), p53 (P00059), and p53 feedback loop (P04398) pathways. In contrast, the same number of genes were differentially expressed in EGF receptor signaling (P00018) in both 2-D and 3-D cultured cells.

These results show enrichment of GO terms was primarily associated with the regulation of gene expression via chromatin, transcriptional, and translational modification, as well as the organization of the ECM/cytoskeleton. Furthermore, 3-D culture conditions appeared to modulate transcriptional regulation of genes associated with several signaling pathways, including Wnt, integrin, cadherin, cytoskeletal regulation by Rho GTPase, and p53.

### 3.4. KEGG Pathway Analysis of Naïve ESCs Grown in 2-D and 3-D Culture Conditions

**Figure 6:**
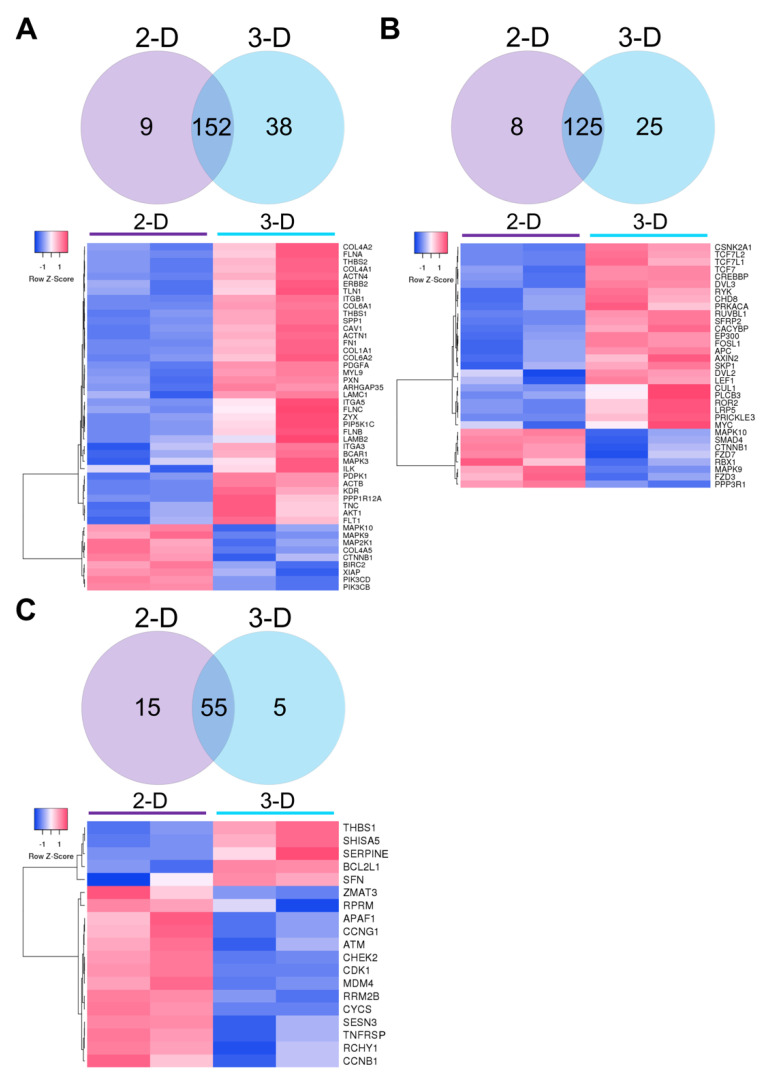
Heatmaps showing raw z-scores of RNA-seq log2 transformed values of 2-D and 3-D grown naïve ESCs. Venn diagrams show the number of genes upregulated in 2-D, genes not significantly changed between 2-D and 3-D, and genes upregulated in Elf1 cells during growth in 3-D scaffolds. Up- and downregulated genes are red and blue, respectively, at a cutoff of *p* < 0.05. Heatmaps depicting significantly DEGs associated with the (**A**) focal adhesion pathway, (**B**) Wnt pathway, and (**C**) p53 pathway were determined using Kyoto Encyclopedia of Genes and Genomes (KEGG) analysis.

We then examined the most significantly enriched pathways in cells grown in 2-D and 3-D culture conditions. DEGs were mapped to the KEGG database (*p* < 0.05; FDR < 0.25) in focal adhesion, Wnt signaling, and p53 signaling pathways and visualized using Venn diagrams and heatmaps (Figure 6). There were 38 genes associated with the focal adhesion pathway upregulated in cells grown in 3-D as opposed to 9 genes in 2-D culture conditions (Figure 6A). Genes upregulated in 3-D grown cells encoded basement membrane proteins (i.e., *COL4A2*, *COL6A1*, *FN1*, *LAMC1*, and *LAMB2*), actin-binding proteins (i.e., *ACTN4*, *FLNA*, *FLNC*, and *TLN1*), as well as components of the integrin signaling pathway (i.e., *CAV1*, *ITGB1*, *ITGA3*, *ITGA5*, and *PXN*), and downstream kinases (i.e., *AKT1* and *MAPK3*). Twenty-five genes involved in the Wnt signaling pathway were enriched in 3-D grown cells (i.e., *TCF7L2*, *TC7L1*, *TCF7*, *CREBBP*, *DVL3*, *RUVBL1*, *SFRP2*, *FOSL1*, *APC*, *AXIN2*, *LEF1*, *LRP5*, and *MYC*) in comparison to only 8 upregulated genes observed in 2-D grown cells (i.e., *MAPK10*, *SMAD4*, *FZD7*, and *FZD3*) (Figure 6B). In contrast, only 5 genes of the p53 pathway were upregulated in 3-D culture (i.e., *THBS1*, *SHISA5*, *SERPINE*, *BCL2L1*, and *SFN*), compared to the 15 upregulated genes (i.e., *RPRM*, *CCNG1*, *APAF1*, *ATM*, *CHEK2*, *CDK1*, *MDM4*, and *CYCS*) represented in 2-D grown cells (Figure 6C). Generally, KEGG analysis of enriched pathways suggested that focal adhesion and Wnt pathways were upregulated, while p53 pathway signaling was downregulated upon the 3-D culture of Elf1 cells.

### 3.5. Validation of RNA-seq Results with qRT–PCR

**Figure 7:**
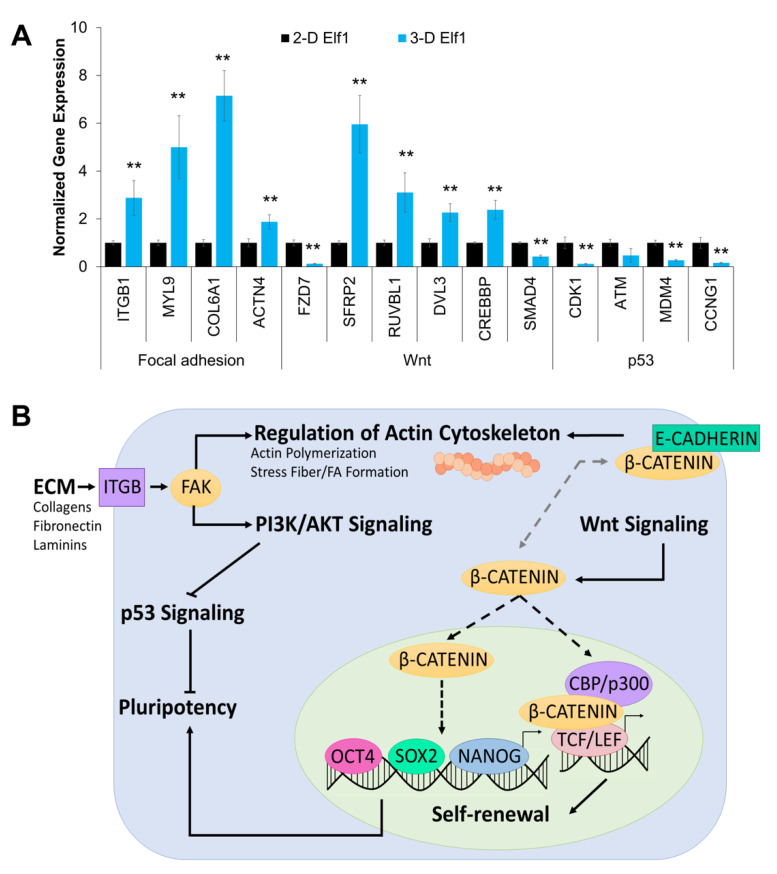
Analysis of genes involved in the maintenance of pluripotency in 3-D grown naïve ESCs. (**A**) 3-D culture conditions affected the expression of genes associated with focal adhesion, Wnt, and p53 signaling pathways as determined by qRT–PCR. Results were expressed as the fold expression ± SEM normalized to reference genes *HMBS* and *GAPDH* (** *p* < 0.01). (**B**) The proposed pathway involved in the maintenance of pluripotency of Elf1 cells grown under 3-D culture conditions.

We next confirmed the RNA-seq data by analyzing selected genes representing various differentially expressed pathways by qRT–PCR (Figure 7). Consistent with the RNA-seq data, the genes associated with focal adhesion, including *ITGB1, MYL9, COL6A1*, and *ACTN4,* were significantly increased in 3-D relative to 2-D grown cells. Similarly, within the Wnt signaling pathway, *SFRP2*, *RUVBL1, DVL3*, and *CREBBP*, were upregulated and *FZD7* and *SMAD4* were downregulated in cells grown in 3-D compared to 2-D culture conditions. On the other hand, *CDK1*, *MDM4*, and *CCNG1* genes, associated with the p53 pathway, were downregulated in cells grown in.

3-D is relative to 2-D culture. The trend in the expression of these genes is consistent with what was observed in the RNA-seq data. Taken together, these results lead us to propose a molecular mechanism involved in the self-renewal and pluripotency of Elf1 cells in 3-D culture, as depicted in Figure 7B.

## 4. Discussion

The two-dimensional culture of human ESCs is marred by batch-to-batch variation, poor viability, and spontaneous differentiation [27,28]. It is well-recognized that cues from culture conditions, including media composition and substrate stiffness, influence self-renewal and cell fate [24]. To circumvent these problems, we investigated the effect of 3-D culture on the growth, maintenance, and gene expression of naïve human ESCs.

Elf1 cells grown in scaffolds composed of self-assembling PEG polymers for three weeks without passaging maintained their pluripotent properties, including self-renewal and differentiation potential, as evident by their ability to form teratomas. The growth of cells in the 3-D scaffolds produced darker and more compact colonies compared to cells cultured under 2-D conditions. Interestingly, a more rapid increase in colony size was observed between days 1 and 14, with a more gradual increase in the average diameter of colonies observed between days 14 and 21 of culture. This is consistent with our previous studies, which showed that encapsulated cell growth was proportional to the rate of scaffold swelling/degradation [36,37]. Furthermore, hydrogel scaffold degradation via hydrolysis over time has been shown to lead to changes in the mechanical properties of the scaffolds [52]. As such, the mechanical strength and dimensions of the scaffolds play important roles in both the restriction of colony size and the rate of cell proliferation. Interactions between cells and their 3-D microenvironment is also an important determinant of cell fate. In the absence of biomaterials or external agitation, ESCs rapidly aggregate to form 3-D spheroids or embryoid bodies (EBs) ranging approximately 100–400 µm in suspension culture [53]. Upon formation, EBs have been shown to mimic the developing embryo, allowing for directed differentiation of cells, which can be controlled by various factors, including EB size [53,54,55,56,57]. In contrast, Elf1 cells encapsulated in the self-assembling scaffolds maintained their naïve pluripotent state, with increased expression of the core as well as naïve pluripotent markers observed in 3-D grown cells. However, their expression declined to normal levels when subcultured back to 2-D culture conditions, suggesting that 3-D culture conditions were responsible for the modulation of gene expression.

The upregulation of pluripotent markers observed during 3-D culture is likely due to numerous factors, including both physical and chemical properties of the self-assembling scaffolds. Stem cell maintenance and differentiation have been shown to be affected by the incorporation of biomaterials as well as scaffold biodegradability, mechanical strength, stiffness, and porosity [58,59]. Although natural biomaterials have been shown to induce biological signaling [34], studies utilizing 3-D scaffolds comprised of hyaluronic acid [30], alginate, and chitosan [60] promoted maintenance of human primed ESC pluripotency without a significant change in *OCT4* marker expression. These findings may be due to the inherited variability associated with natural biomaterials or due to their complicated and pleiotropic roles in cell growth. In a similar study, human ESCs were passaged over 60 times using thermoresponsive synthetic scaffolds comprised of PEG functionalized with poly-N-isopropylacrylamide, supporting the maintenance of primed pluripotency with 95% *OCT4* positive cells [61].

In contrast, alteration of scaffold stiffness has been shown to modulate gene expression with upregulation of select core pluripotent markers observed in the 3-D culture of mouse [36,62] as well as human ESCs [63,64], suggesting an important role of the mechanical microenvironment. Furthermore, we have reported improved growth and upregulation of core and select naïve pluripotent markers in primed human ESCs in self-assembling scaffolds comprised of PEG-8-SH/PEG-8-Acr, when compared to scaffolds prepared with lower crosslinking densities [37]. In addition to their more controllable mechanical properties, the synthetic nature of PEG scaffolds allows for the ability to decouple the complex effect of biological and mechanical signaling. To determine the influence of the 3-D culture microenvironment on gene expression profiles in Elf1 cells, we performed a global transcriptomic analysis.

We identified a significant number of DEGs in 2-D and 3-D culture conditions. Functional analysis of these genes and overlap of GO terms indicated that 3-D culture conditions impacted the regulation of transcription and chromatin as well as the organization of the ECM, modulated via various signaling pathways. 3-D grown cells also displayed enrichment of biological process GO terms, including mRNA processing, mRNA stability, and translation, which could be responsible for the long-term self-renewal observed upon encapsulation in the self-assembling scaffold. We also observed upregulated GO terms associated with chromatin remodeling and histone methyltransferase activity in 3-D grown naïve ESCs, shown to be involved with increased transcriptional activation [65] and the establishment of induced pluripotency [66]. Upregulation of genes associated with chromatin remodeling in 3-D grown cells may suggest a continued open chromatin structure needed for the genes involved in pluripotency. This would be consistent with the higher expression of many of the observed pluripotent markers. Previous RNA-seq studies comparing naïve and primed human ESC epigenomic dynamics suggest that a more open chromatin structure is associated with naïve pluripotency, with enhancer chromatin and 3-D genome architecture changes reported to be involved in the transition from naïve to primed ESCs [67]. This may imply that the 3-D scaffold environment was more supportive of the maintenance of the naïve pluripotent state.

Extensive overlap of GO terms associated with the organization of the ECM and modulation of the actin cytoskeleton were also significantly overrepresented in 3-D grown cells. 3-D grown cells exhibited increased DEGs encoding major components of the basement membrane synthesized by pluripotent stem cells, including collagen, fibronectin, and laminin, which have been shown to support ESC pluripotency when used as 2-D culture substrates via integrin binding [68]. It is well-known that the transduction of biophysical cues from the microenvironment, including matrix dimensionality, mechanical forces, and cell–cell interactions, can influence ESC shape and fate determination as well as ECM deposition during development [25].

Cell interaction with the surrounding ECM can affect the cytoskeletal organization, nuclear morphology, and in turn, can affect gene expression via alteration of actin fibers [69]. In our study, 3-D grown cells exhibited clonal growth forming large rounded colonies during growth in the self-assembling scaffold and upregulation of DEGs associated with actin cytoskeleton remodeling while maintaining pluripotency. In a recent study, 2-D maintenance of human ESCs using various growth media was shown to differentially influence cytoskeleton protein expression and morphology in a cell-line-dependent manner, with culture in more defined media resulting in more compact clonal growth [70]. Since we used the same growth media for both 2-D and 3-D culture, it is plausible that the dimensionality and/or composition of the scaffold was responsible for the observed transcriptional changes associated with ECM and cytoskeletal organization. Furthermore, since the PEG-8-SH/PEG-8-Acr scaffold is biologically inert, the 3-D scaffold provided a more defined microenvironment, which altered mechanical cues typically associated with 2-D culture.

PANTHER pathway analysis of 3-D grown Elf1 cells depicted a higher representation of DEGs associated with cytoskeleton regulation by Rho GTPase as well as integrin, cadherin and Wnt signaling; whereas, fewer DEGs were observed in Ras and p53 pathways in comparison to 2-D cultured cells. Based on these findings, we performed further KEGG pathway analyses and proposed a potential mechanism for the long-term maintenance of naïve pluripotency in Elf1 cells grown in the 3-D self-assembling scaffolds.

In both mouse and human ESCs, focal adhesion, and integrin signaling serve pleiotropic roles in anchorage, contractility, survival, proliferation, as well as support or inhibition of self-renewal [71]. Our study revealed upregulation in 3-D grown cells of several genes such as *FLNC*, *COL4*, *CAV1*, and *SERPINE1*, as well as *ITGB1*, *ILK*, and *AKT1*, which have been reported to be associated with actin cytoskeleton remodeling [72], and integrin-mediated focal adhesion activation [73], respectively. Activated integrin signaling stimulates the PI3K/Akt signaling pathway, which results in downstream inhibition of the ERK signaling pathway [74], playing an essential role in both mouse and human ESC pluripotency [75]. Additionally, mechanical cues arising from the ECM are transduced to the nucleus through cytoskeleton remodeling and stress fibers via integrins and focal adhesion complexes [76]. Changes in the actin cytoskeleton induced by substrate stiffness are often regulated by activation of Rho GTPases [77] and promote translocation of mechanosensitive transcription factors such as Yes-associated protein (YAP) to the nucleus in human ESCs [78]. Induction of YAP expression was shown to aid in the long-term survival and expansion of human ESCs in 2-D culture [79]. Furthermore, we have shown the induction of naïve-like pluripotency in primed human ESCs grown in 3-D culture, which was abolished upon inhibition of YAP [37]. These observations further suggest a mechanistic role of cytoskeleton remodeling and mechanical signaling in the maintenance of pluripotency.

In addition, we observed differential activation of Wnt signaling in 2-D and 3-D culture conditions at a transcriptional level, which is supported by several studies suggesting that application of mechanical stimuli and alteration in substrate topography can stimulate Wnt signaling, which plays a role in cell differentiation and maturation in adult stem cells [80,81,82]. In ESCs, Wnt/β-catenin signaling performs functions in self-renewal, differentiation, and lineage commitment [83]. Activation of Wnt signaling has been shown to promote self-renewal of naïve human ESCs but is not directly required for the expression of pluripotent markers [84]. Upon transition from the naïve to the primed state, Wnt signaling is largely downregulated [85]. Furthermore, sustained activation of Wnt/β-catenin signaling is needed to preserve normal DNA methylation epigenetic stability in mouse ESCs [86]. Our results suggest diverse transcriptional changes associated with the Wnt pathway, including upregulation of downstream Wnt targets, including transcription factors, *TCF7*, *TCF7L1*, and *LEF1*, and chromatin remodeling complexes *CREBBP* and *EP300*, encoding CREB-binding protein (CBP) and p300, respectively. While TCF7 has been shown to be essential for self-renewal in mouse ESCs [87], TCF7L1 plays an important role in human ESC pluripotency via suppression of primitive streak gene expression [88]. Overexpression of TCF7L1 causes downregulation of mesodermal gene expression and leads to an improved somatic cell reprogramming and colony formation [89]. However, stabilization of β-catenin has been shown to also enhance self-renewal in naïve mouse ESCs in a TCF independent manner, whereby nuclear β-catenin acts by directly interacting with the master transcription factor, OCT4, to induce the expression of pluripotent genes [90]. Coactivators p300/CBP serve redundant roles to stabilize long-range chromatin loops, which aid in the recruitment of other transcription factors and coregulators, thus promoting the expression of self-renewal genes [91]. Furthermore, transcriptional coactivator p300 has been shown to directly aid in the recruitment of the NANOG-OCT4-SOX2 complex in mouse ESCs [92].

Interestingly, human ESC self-renewal has also been shown to be enhanced by short-term stimulation of Wnt/β-catenin signaling via upregulation of E-cadherin and the subsequent activation of the PI3K/Akt pathway [83]. We observed a higher number of upregulated DEGs associated with cadherin signaling in 3-D grown cells. This may suggest a role for the cadherin-mediated cell-cell interactions in the maintenance of the self-renewing state via altered cell-cell interactions in 3-D culture. E-cadherin has been shown to stabilize LIF signaling [93] and control intracellular levels of β-catenin [94]. Furthermore, 2-D grown cells were characterized by upregulated DEGs in the Ras signaling pathway. Ras signaling is repressed in naïve pluripotency and modulates cadherin protein expression and the epigenetic landscape, playing an important role in the transition to primed pluripotency [95]. We observed the downregulation of *CTNNB1*, the gene encoding β-catenin, in 3-D grown cells. Since Wnt signaling is regulated by post-translational modifications of β-catenin and extensive crosstalk with other signaling pathways [96], additional studies are warranted to determine the levels of activated β-catenin upon culture in 3-D scaffolds.

Global transcriptome analysis suggested that 3-D culture in the self-assembling scaffold repressed the p53 signaling pathway in Elf1 cells. p53 acts as a transcriptional regulator of cell cycle arrest and apoptosis and is important for the maintenance of genetic integrity in response to various cellular stresses [97]. In ESCs, p53 is present at low levels in the nucleus, and activation is associated with the induction of differentiation, repressing self-renewal via regulation of key pluripotent markers and microRNAs [98,99,100]. As such, both the maintenance of pluripotency and efficient somatic reprogramming require the suppression of p53 signaling [99]. Additionally, integrin-mediation via focal adhesion kinase (FAK) stimulates Akt signaling, resulting in downstream suppression of p53, which promotes cell survival and maintenance of pluripotency [101]. Transcriptional comparison of naïve ESCs showed increased expression of DEGs associated with the p53 pathway and the p53 feedback regulatory loop, involving *APAF1*, *ATM*, and *CYCS*, when cells were cultured in 2-D culture conditions. This may indicate that 2-D culture resulted in increased heterogeneity due to spontaneous differentiation [27] or enhanced proliferation [102], well-known limitations of traditional planar culture [6].

## 5. Conclusions

Overall, 3-D culture conditions appeared to modulate transcriptional regulation of several signaling pathways associated with adhesion and self-renewal, implicating that transduction of mechanical cues from the surrounding scaffold microenvironment may play a role in the regulation of self-renewal and pluripotent marker expression in 3-D culture conditions.

Although these results based on global gene expression are interesting, further studies are warranted that focus on determining the effect of mechanical stress on cytoskeleton dynamics, as well as activation of Wnt and p53 signaling at a translational level. Additional knockdown studies for controlled Wnt and/or p53 signaling may also provide useful insights into the regulation of naïve and primed pluripotent states. It would also be interesting to delineate the effects of cell-cell and cell-material mechanical interactions by comparing the effect of comparatively sized ESC spheroids on gene expression. Nevertheless, our findings suggest the importance of mechanical signaling and culture dimensionality on ESC self-renewal and may foster further developments in the long-term 3-D culture of ESCs and their use for cell therapy and regenerative medicine.

## Figures and Tables

**Table 1:** List of human primer sequences used in qRT–PCR.

Gene	Primer Sequence		
	Forward (5′-3′)	Reverse (5′-3′)	Product Length
*ACTN4*	TGGCTGCTGAATGAGATCCG	GGCTTTGATGTCCGATAGTGT	156
*ATM*	CCTACCAAATCCCTCCACC	CCTTGAGCATCCCTTGTGTT	236
*CCNG1*	AATGAAGGTACAGCCCAAGCA	GCTTTGACTTTCCAACACACC	195
*CDK1*	TGCTGGGGTCAGCTCGTTAC	TGGGATGCTAGGCTTCCTGG	232
*COL6A1*	TCAAGAGCCTGCAGTGGATG	TGGACACTTCTTGTCTATGC	377
*CREBBP*	AGTAACGGCACAGCCTCTCA	CCTGTCGATACAGTGCTTCTAG	115
*DNMT3L*	CTGCTCCATCTGCTGCTCC	ATCCACACACTCGAAGCAGT	85
*DPPA3*	AGACCAACAAACAAGGAGCCT	CCCATCCATTAGACACGCAGA	88
*DVL3*	ACAATGCCAAGCTACCATGCTTC	AGCTCCGATGGGTTATCAGCAC	103
*FZD7*	GCAAAGCAGCGCAAATCTGA	AACCTCTGGCTTAACGGTGTGTG	116
*GAPDH*	ACAACTTTGGTATCGTGGAAGG	GCCATCACGCCACAGTTTC	101
*HMBS*	AGGAGTTCAGTGCCATCATCCT	CACAGCATACATGCATTCCTCA	104
*ITGB1*	GGATTCTCCAGAAGGTGGTTT	TGCCACCAAGTTTCCCATCT	143
*KLF17*	TCAGGAAGGGACTGGTAGAA	GTACCCGCATATGTCGTCTAAG	206
*KLF4*	CGAACCCACACAGGTGAGAA	TACGGTAGTGCCTGGTCAGTTC	75
*MDM4*	CTAAGTCCTTAAGTGATGATACCGATG	AACTTTGAACAATCTGAATACCAATCC	151
*MIXL1*	CCGAGTCCAGGATCCAGGTA	CTCTGACGCCGAGACTTGG	58
*MYL9*	GAGCCCAAGCGCCTTCT	GTCAATGAAGCCATCACGGT	202
*NANOG*	AAAGAATCTTCACCTATGCC	GAAGGAAGAGGAGAGACAGT	110
*N-CADHERIN*	TGTTTGGCCTGGCGTTCTTT	AGGAGACAGAAACGAAGCCA	156
*NCAM*	AGGAGACAGAAACGAAGCCA	GGTGTTGGAAATGCTCTGGT	161
*OCT4*	CCCCTGGTGCCGTGAA	GCAAATTGCTCGAGTTCTTTCTG	97
*PAX6*	CTTTGCTTGGGAAATCCGAG	AGCCAGGTTGCGAAGAACTC	103
*RUVBL1*	TTGCTCAGGAGCTGGGTAGT	CCCATGGGATTCTCTGTCTC	196
*sFRP2*	ACGGCATCGAATACCAGAACA	CTCGTCTAGGTCATCGAGGCA	176
*SMAD4*	CCAGGATCAGTAGGTGGAAT	GTCTAAAGGTTGTGGGTCTG	243
*SOX17*	CGCACGGAATTTGAACAGTA	GGATCAGGGACCTGTCACAC	182
*SOX2*	TTGCTGCCTCTTTAAGACTAGGA	CTGGGGCTCAAACTTCTCTC	75
*SOX7*	ACGCCGAGCTCAGCAAGAT	TCCACGTACGGCCTCTTCTG	73
*TFCP2L1*	TTTGTGGGACCCTGCGAAG	TGCTTAAACGTGTCAATCTGGA	129

## Data Availability

Publicly available datasets were analyzed in this study. This data can be found here: https://www.ncbi.nlm.nih.gov/geo/query/acc.cgi?acc=GSE161306.
